# Precise Orbit Solution for Swarm Using Space-Borne GPS Data and Optimized Pseudo-Stochastic Pulses

**DOI:** 10.3390/s17030635

**Published:** 2017-03-20

**Authors:** Bingbing Zhang, Zhengtao Wang, Lv Zhou, Jiandi Feng, Yaodong Qiu, Fupeng Li

**Affiliations:** 1 School of Geodesy and Geomatics, Wuhan University, Wuhan 430079, China; bbzhang@whu.edu.cn (B.Z.); zhoulv@whu.edu.cn (L.Z.); jdfeng@whu.edu.cn (J.F.); ydqiu@whu.edu.cn (Y.Q.); fpli@whu.edu.cn (F.L.); 2 Beijing Key Laboratory of Urban Spatial Information Engineering, Beijing Institute of Surveying and Mapping, Beijing 100038, China

**Keywords:** Swarm, precise orbit determination, reduced-dynamic method, space-borne dual-frequency GPS data, pseudo-stochastic pulses, orbit accuracy

## Abstract

Swarm is a European Space Agency (ESA) project that was launched on 22 November 2013, which consists of three Swarm satellites. Swarm precise orbits are essential to the success of the above project. This study investigates how well Swarm zero-differenced (ZD) reduced-dynamic orbit solutions can be determined using space-borne GPS data and optimized pseudo-stochastic pulses under high ionospheric activity. We choose Swarm space-borne GPS data from 1–25 October 2014, and Swarm reduced-dynamic orbits are obtained. Orbit quality is assessed by GPS phase observation residuals and compared with Precise Science Orbits (PSOs) released by ESA. Results show that pseudo-stochastic pulses with a time interval of 6 min and a priori standard deviation (STD) of 10^−2^ mm/s in radial (R), along-track (T) and cross-track (N) directions are optimized to Swarm ZD reduced-dynamic precise orbit determination (POD). During high ionospheric activity, the mean Root Mean Square (RMS) of Swarm GPS phase residuals is at 9–11 mm, Swarm orbit solutions are also compared with Swarm PSOs released by ESA and the accuracy of Swarm orbits can reach 2–4 cm in R, T and N directions. Independent Satellite Laser Ranging (SLR) validation indicates that Swarm reduced-dynamic orbits have an accuracy of 2–4 cm. Swarm-B orbit quality is better than those of Swarm-A and Swarm-C. The Swarm orbits can be applied to the geomagnetic, geoelectric and gravity field recovery.

## 1. Introduction

Swarm is a European Space Agency (ESA) project that was launched on 22 November 2013, which consists of three Swarm satellites. Swarm-A and Swarm-C are flying at orbital altitude of 480 km, Swarm-B is flying at orbital altitude of 530 km. All three Swarm satellites carry advanced scientific equipment to achieve the project’s primary objective [1,2,3], which is to investigate the mechanism of the geomagnetic field [3]. The secondary objective is to monitor the change of Earth’s gravity field via the on-board GPS tracking of Swarm [2]. Vector and scalar magnetometers were used on the Swarm satellites to monitor the change of the geomagnetic field; a thermal ion imager and a Langmuir probe were applied to monitor the geoelectric field information; space-borne GPS receivers were used to research the precise orbit determination (POD) and laser retro-reflectors were applied to validate Swarm POD [2,3].

Given the successful use of space-borne GPS POD, more satellites are expected to carry space-borne GPS receivers to fulfill their orbit accuracy requirements, which may range from one centimeter to hundreds of meters [4,5,6,7,8,9,10,11,12,13,14]. Geodetic and oceanographic satellites (e.g., HY2A, Challenging Minisatellite Payload (CHAMP), Gravity Recovery and Climate Experiment (GRACE), Gravity field and steady-state Ocean Circulation Explorer (GOCE), Constellation Observation System for Meteorology, Ionosphere and Climate (COSMIC) and Swarm) have more stringent orbit accuracy requirements. Swarm space-borne GPS receivers are produced by Rüstungs Unternehmen Aktiengesellschaft Space, which are used to make tracks for GPS satellite signal [15]. All three Swarm satellites are equipped with space-borne dual-frequency GPS receivers.

Swarm precise orbits can be applied to the geomagnetic field, geoelectric field and the recovery of the Earth’s static and time-variable gravity field. Thus, after the launch of Swarm satellites, some scholars have studied Swarm POD using on-board GPS data. For example, van den IJssel et al. [3] used GPS High precision Orbit determination Software Tools (GHOST) to calculate Swarm zero-differenced reduced-dynamic POD and kinematic POD, the consistency between the two orbits is at 4–5 cm. Furthermore, Jäggi et al. [2] computed the reduced-dynamic orbits and kinematic orbits by Bernese Global Navigation Satellite System (GNSS) Software and then used 18 months of Swarm kinematic orbits to calculate the Earth gravity field model. The above studies introduce empirical accelerations to absorb force modeling errors and deficiencies based on the zero-differenced (ZD) reduced-dynamic technique. However, few studies are conducted on pseudo-stochastic pulses used in Swarm ZD reduced-dynamic orbit solutions. In addition, ESA released a four-day delay in Swarm on-board GPS observations, whereas Swarm Precise Science Orbits (PSOs) had a delay of three weeks. To obtain Swarm precise orbits in time, autonomous orbit determination is important to carry out follow-up research work (e.g., Earth gravity field and geomagnetic field research). Thus, it is necessary to study Swarm precise orbit solutions.

We study the orbit quality that Swarm orbits can achieve under high ionospheric activity by using optimized pseudo-stochastic pulses based on the ZD reduced-dynamic technique. During the process, space-borne dual-frequency GPS observations and suitable force models are used, especially with the aid of a highly advanced gravity field model, EIGEN-6S4 [16].

This article presents a new POD strategy for all three Swarm satellites using the space-borne dual-frequency GPS data together with reduced dynamic models. The study computes Swarm precise orbits via a ZD reduced-dynamic approach based on Bernese software. The approach combines space-borne GPS observation from Swarm level 1B products released by ESA [17] and suitable force models. The optimized pseudo-stochastic pulses are introduced into the Swarm satellite’s deterministic motion equation. Orbit quality is evaluated by GPS phase residuals and comparison with PSOs released by ESA [10].

This article consists of the following sections. Section 2 introduces three main approaches for Low Earth Orbiter (LEO) precise orbit solutions. Force models, GPS observations and estimated parameters for Swarm Orbit Determination (OD) are introduced in detail. Section 3 presents the data processing strategy. Section 4 introduces the relevant experiments. Conclusions are showed in Section 5.

## 2. Reduced Dynamic Orbit Determination

Space-borne GPS for Low Earth Orbiter (LEO) POD is the most effective technology that offers continuous space-borne GPS data. There are three main approaches to LEO precise orbit solutions making use of space-borne GPS observation. The first approach is pure dynamic OD [18,19], which depends on highly precise force models and corrects a few dynamic parameters as part of the LEO POD. The second approach is kinematic OD [20], which is different from satellite dynamics because the kinematic orbit solution process demands only pure geometric information offered by the GPS data. The third approach is reduced-dynamic OD [21,22,23,24,25,26], which makes the best of the dynamic and geometric information. The outstanding reduced-dynamic approach estimates the pseudo-stochastic parameters [27] (e.g., pseudo-stochastic pulses and empirical accelerations) to absorb force model errors.

The outstanding reduced-dynamic approach is chosen for Swarm POD. Swarm orbit strategy is presented in detail in the following section. The orbit quality of Swarm satellite in the ZD reduced-dynamic POD depends on the quality of force models, the POD strategies (i.e., pseudo-stochastic pulses optimization), and the quality of GPS observations applied to the ZD reduced-dynamic solutions. Proper force models should be selected for precise orbit determination of Swarm satellites. In this paper, some considered force models include gravity field model EIGEN-6S4, solid tide model IERS2010, and tide model FES2004. Satellite attitude control file comes from the ESA. Solar pressure and other unmodeled force errors are replaced by pseudo-stochastic pulse parameters. During the LEO orbit determination, it is not necessary to set stochastic velocity pulses in each observation epoch, but rather to set one or a set of stochastic pulses at a certain time interval, so it is called pseudo-stochastic pulses. The velocity variations can be constrained to suitably small values by means of pseudo-observed values of the velocity change introduced in advance [22]. Therefore, pseudo-stochastic parameters are expressed by pseudo-stochastic pulses in R, T and N directions with a time interval and a priori standard deviation (STD). Table 1 shows the model standards adopted for Swarm ZD reduced-dynamic POD.

## 3. Swarm Orbit Strategy

ZD reduced-dynamic method is chosen in this article. Given that high-precise clock products and GPS orbits are essential, we choose 5 s rate clock data [31] and final GPS ephemeris [32], which are offered by the Center for Orbit Determination in Europe (CODE) Analysis Center. EIGEN-6S4 is a high-quality gravity field model which is used to improve the accuracy of a gravity field model in the Swarm ZD POD. Swarm ZD GPS code and phase observations with 10 s sampling interval are presented. A series of measures are used to select high-quality on-board GPS observation. For example, the elevation cut-off angle is set at 3°, and the signal-to-noise ratio is set as a threshold of 5. Moreover, high ionospheric activity can easily lead to cycle slips. Thus, in order to better detect and repair cycle slips and provide good GPS data for improving Swarm orbits, we choose TurboEdit and COMBINED algorithm, which are widely used in LEO space-borne dual-frequency GPS data [33,34]. A development orbit strategy of the ZD reduced-dynamic approach is applied to estimate the orbit parameters and relevant parameters (e.g., the values of phase ambiguities and receiver clock error corrections). Pseudo-stochastic parameters are expressed by pseudo-stochastic pulses in radial (R), along-track (T) and cross-track (N) directions with a time interval and a priori STD.

Many scholars have conducted research on the reduced-dynamic POD of LEO: CHAMP, GRACE and GOCE. Pseudo-stochastic pulses are derived from experience. For example, under the condition that the time interval is 15 min and a priori STD is 10^−3^ mm/s, the space environment of each LEO is different, applying previous empirical values to Swarm ZD reduced-dynamic POD may not obtain the best orbit solution. Therefore, the optimized pseudo-stochastic pulses of in-depth analysis and research are important in this article. Here, we offer a set of optimized empirical values to obtain better orbit solutions.

Based on the following two considerations, Swarm PSOs released by ESA are considered as reference orbits in the paper. On one hand, Swarm PSOs released by ESA used piecewise constant acceleration in a reduced-dynamic orbit determination approach. Independent satellite laser ranging validation indicates that Swarm PSOs have accuracies over 2 cm [3]. Therefore, ESA released reliable Swarm PSOs. In the article, Swarm PSOs released by ESA are compared and analyzed. On the other hand, results of Swarm orbit accuracy are analyzed comprehensively from radial, along-track, cross-track and 3D directions. Therefore, results are compared with Swarm PSOs released by ESA to carry out follow-up related research.

Thus, in order to evaluate orbit quality, Swarm orbit solutions are compared with Swarm PSOs released by ESA. In addition, Swarm GPS phase residuals permit the quality of the force models and observation models. Root Mean Square (RMS) of Swarm GPS phase residuals are at the level of data precision if the force models and observations are modeled perfectly [10].

Pseudo-stochastic pulse parameter is determined using a priori standard deviation (STD) and time interval. So, pseudo-stochastic pulse parameter optimization can effectively absorb force model and modeled mechanical error, thus improving orbit determination accuracy. Therefore, we obtain a set of optimized pseudo-stochastic pulses by analyzing Swarm GPS phase residuals and orbit differences. The relevant experiments are shown below.

For time interval, relevant experts conducted corresponding research. Jäggi et al. [22] discussed that a time interval of 6 min was proper, two main reasons support this view. One is that filtering effect of LEO orbits is well by a time interval of 6 min. Another is that an interval of 6 min is short enough to reduce the orbit differences of LEO short-periodic variation. The time interval of 6 min is chosen in the article to maintain continuity.

For a priori STD, when a priori STD is too large for pseudo-stochastic pulse, the filter cannot fully use the observed information. When a priori STD is too small, owing to the recursive nature of the filter, model error significantly increases. More serious change possibly leads to filter divergence. Therefore, a priori STD is set as follows from Experiments (1) to (6).

(1) A priori STD is 10^1^ mm/s
(2) A priori STD is 10^0^ mm/s
(3) A priori STD is 10^−1^ mm/s
(4) A priori STD is 10^−2^ mm/s
(5) A priori STD is 10^−3^ mm/s
(6) A priori STD is 10^−4^ mm/s

The orbit arc length is usually 24 h, which is equivalent to one full day. Therefore, we randomly selected data on 1 November 2014, to participate in the Swarm ZD reduced-dynamic POD and better understand the effect of pseudo-stochastic pulses on the Swarm ZD reduced-dynamic POD. The conclusion is applicable to orbit arc length of 24 h.

**Figure 1:**
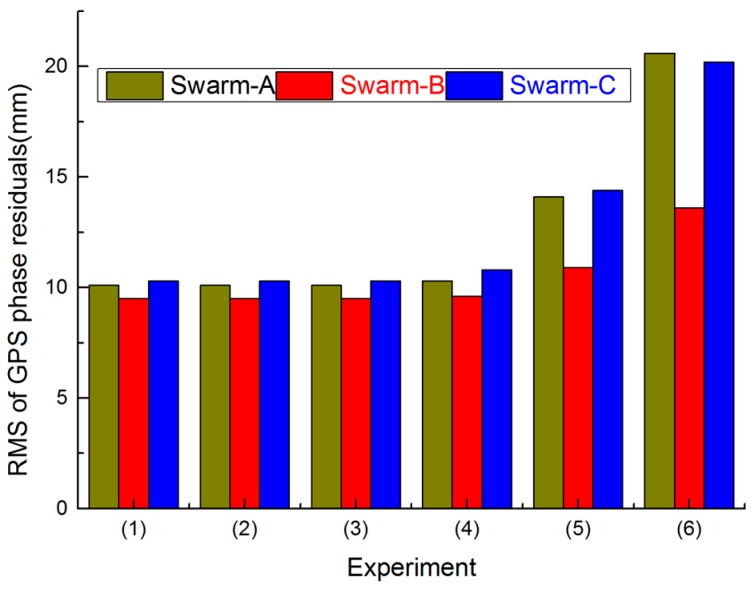
Root Mean Square (RMS) values of GPS phase residuals for all three Swarm satellites on 1 November 2014.

Figure 1 indicates the RMS values of GPS phase residuals for all three Swarm satellites from the above six experiments. The RMS values of GPS phase residuals from Experiments (1) to (4) are basically the same; however, the RMS values of GPS phase observation residuals from Experiments (5) to (6) are rapidly increasing.

**Figure 2:**
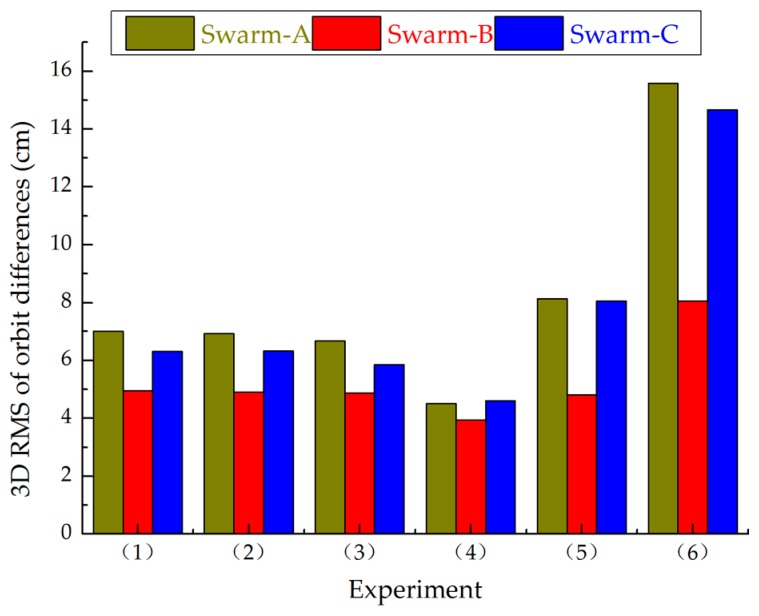
RMS values of orbit differences for all Swarm satellites in 3D direction between Swarm POD and Precise Science Orbits (PSOs) released by the European Space Agency (ESA), 1 November 2014.

Figure 2 shows the RMS values of orbit differences for all Swarm satellites in 3D direction between Swarm POD and PSOs released by ESA on 1 November 2014: $3D=\sqrt{R^{2}+T^{2}+N^{2}}$. The RMS values of orbit differences for Swarm in 3D direction from Experiments (1) to (4) are well and stable. The result is consistent with GPS phase residuals, and the only difference is that the RMS of orbit differences for Swarm in 3D direction from Experiment (4) is the best.

According to Figure 1 and Figure 2, we can conclude that the Swarm orbit solutions are basically the same when a priori STD is better than 10^−2^ mm/s, and the Swarm orbit solutions are worse when a priori STD is less than 10^−2^ mm/s. Moreover, F10.7 of the chosen day is 118.1 sfu, which indicates that ionospheric activity is high on the chosen day. If pseudo-stochastic pulses are not optimized (e.g., a priori STD is less than 10^−2^ mm/s), Swarm ZD reduced-dynamic POD cannot obtain high-quality precision orbit determination.

The above experiments show that when a priori STD of the pseudo-stochastic pulses is set to 10^−2^ mm/s in R, T and N directions, the Swarm orbit solution is the best. In order to keep the consistency of the experiments, this paper studies the Swarm orbit accuracy when a priori STD of pseudo-stochastic pulses is different settings in different directions on the basis of the above experiments.

Case 1: A priori STD is 10^−2^ mm/s in T and N directions, a priori STD ranges from 10^1^ mm/s to 10^−4^ mm/s in R direction, which are set as follows from Experiments (7) to (12).

(7) A priori STD is 10^1^ mm/s in R direction,
(8) A priori STD is 10^0^ mm/s in R direction,
(9) A priori STD is 10^−1^ mm/s in R direction,
(10) A priori STD is 10^−2^ mm/s in R direction,
(11) A priori STD is 10^−3^ mm/s in R direction,
(12) A priori STD is 10^−4^ mm/s in R direction.

**Figure 3:**
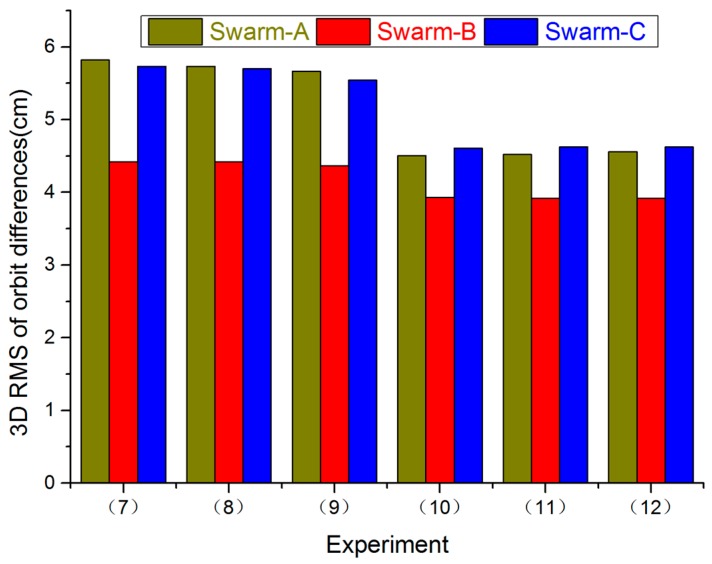
RMS values of orbit differences for all Swarm satellites in 3D direction between Swarm POD and PSOs released by ESA from Experiments (7) to (12), 1 November 2014.

Figure 3 shows RMS values of orbit differences for all Swarm satellites in 3D direction between Swarm POD and PSOs released by ESA from Experiments (7) to (12). The RMS values of orbit differences for Swarm in 3D direction from Experiments (7) to (9) are worse than Experiments (10) to (12). Moreover, Experiments (10) to (12) have the same results. According to Case 1, in R direction, when a priori STD was reduced to 10^−2^ mm/s, orbit accuracy was increased, but when we continued to reduce a priori STD, orbit accuracy was no longer improved.

Case 2: A priori STD is 10^−2^ mm/s in R and N directions, a priori STD ranges from 10^1^ mm/s to 10^−4^ mm/s in T direction, which are set as follows from Experiments (13) to (18).

(13) A priori STD is 10^1^ mm/s in T direction,
(14) A priori STD is 10^0^ mm/s in T direction,
(15) A priori STD is 10^−1^ mm/s in T direction,
(16) A priori STD is 10^−2^ mm/s in T direction,
(17) A priori STD is 10^−3^ mm/s in T direction,
(18) A priori STD is 10^−4^ mm/s in T direction.

**Figure 4:**
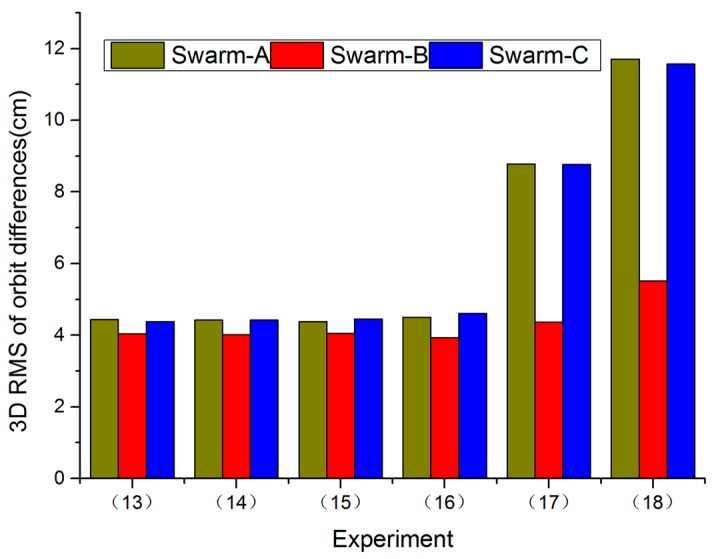
RMS values of orbit differences for all Swarm satellites in 3D direction between Swarm POD and PSOs released by ESA from Experiments (13) to (18), 1 November 2014.

Figure 4 shows RMS values of orbit differences for all Swarm satellites in 3D direction between Swarm POD and PSOs released by ESA from Experiments (13) to (18). The RMS values of orbit differences for Swarm in 3D direction from Experiments (17) to (18) are worse than Experiments (13) to (16). Moreover, Experiments (13) to (16) have the same results. According to Case 2, in T direction, when a priori STD was increased to 10^−2^ mm/s, orbit accuracy was improved rapidly, but when we continued to increase a prior STD, orbit accuracy was no longer improved.

Case 3: A priori STD is 10^−2^ mm/s in R and T directions, a priori STD ranges from 10^1^ mm/s to 10^−4^ mm/s in N direction, which are set as follows from Experiments (19) to (24).

(19) A priori STD is 10^1^ mm/s in N direction,
(20) A priori STD is 10^0^ mm/s in N direction,
(21) A priori STD is 10^−1^ mm/s in N direction,
(22) A priori STD is 10^−2^ mm/s in N direction,
(23) A priori STD is 10^−3^ mm/s in N direction,
(24) A priori STD is 10^−4^ mm/s in N direction.

**Figure 5:**
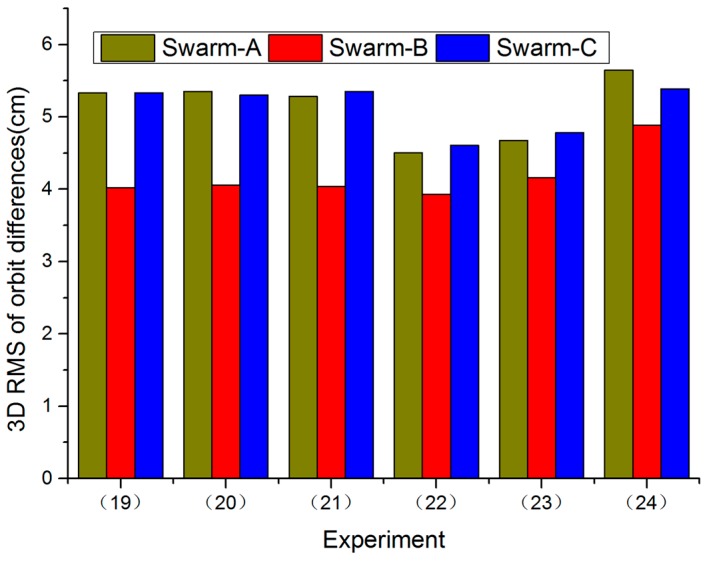
RMS values of orbit differences for all Swarm satellites in 3D direction between Swarm POD and PSOs released by ESA from Experiments (19) to (24), 1 November 2014

Figure 5 shows RMS values of orbit differences for all Swarm satellites in 3D direction between Swarm POD and PSOs released by ESA from Experiments (19) to (24). Experiment (22) has the best result. According to Case 3, in N direction, when a priori STD was reduced to 10^−2^ mm/s, orbit accuracy was improved, but when we continued to reduce a priori STD, orbit accuracy was reduced.

In summary, based on a set of experiments and characteristics of pseudo-stochastic pulse parameters, the present study provides an optimal a priori STD and time interval for pseudo-stochastic pulses. When we choose 24 h arc length to participate in the calculation of Swarm ZD reduced-dynamic POD, pseudo-stochastic pulses in R, T and N directions with a time interval of 6 min and a priori STD of 10^−2^ mm/s are optimized. Therefore, we introduce optimized pseudo-stochastic pulses to compute Swarm ZD reduced-dynamic POD under high ionospheric activity and evaluate the Swarm POD in this study.

## 4. Experiments

The Swarm POD contains two sections. One is computing orbits by the best orbit strategy, and the other is assessing the quality of Swarm orbits. Given that we have not assessed absolute orbit accuracy, we can take advantage of different assessment methods to evaluate the orbit quality (e.g., internal quality assessment and external equality assessment). Two of the widely used methods to evaluate orbit accuracy are GPS phase residuals and independent orbit comparison. Independent orbit comparison contains two sections: orbit comparison between two independent tracking systems and orbit comparison between two different institutions. The assessment methods are successfully applied to CHAMP, GRACE and GOCE. We use GPS phase observation residuals to evaluate internal quality assessment of Swarm orbits. In addition, we use comparison with ESA PSOs and Satellite Laser Ranging (SLR) validation to evaluate external equality assessment.

We validate the effectiveness of the ZD reduced-dynamic method and Swarm orbit strategy (containing optimized pseudo-stochastic pulses) by carrying out related experiments under high ionospheric activity in the following section.

**Figure 6:**
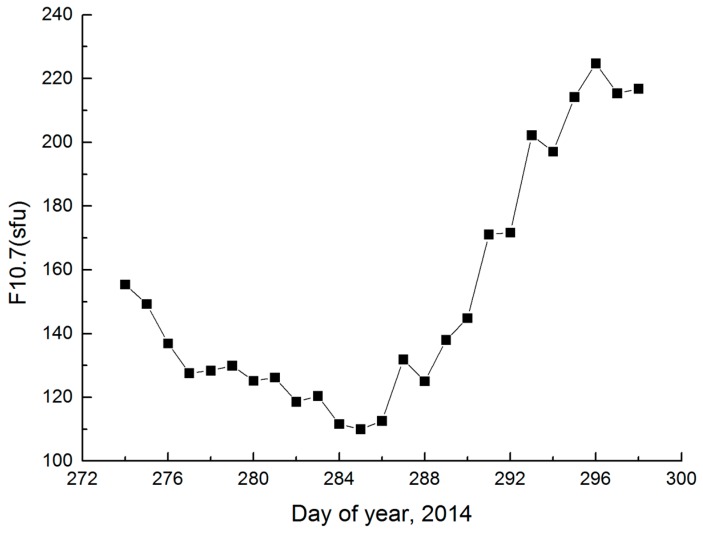
F10.7 values during 1–25 October 2014.

First, we choose 1–25 October 2014 (day of year (DOY) 274 to 298, 2014) of the Swarm space-borne GPS data. Figure 6 shows F10.7 values from 1–25 October 2014. The Figure indicates that F10.7 values vary from 110 sfu to 224.8 sfu (1 sfu = 10^−22^ Wm^−2^·Hz^−1^), which demonstrates that the ionospheric activity is high during this period. The chosen Swarm space-borne GPS data are suitable when conducting research on Swarm ZD reduced-dynamic POD under high ionospheric activity.

Second, we use chose Swarm GPS data to participate in the Swarm ZD reduced-dynamic OD based on Swarm orbit strategy in the article.

Finally, we use GPS phase observation residuals to evaluate the internal quality assessment of Swarm orbits. The external equality assessment is then evaluated by comparison with Swarm PSOs released by ESA and SLR validation.

### 4.1. Swarm GPS Phase Residuals

**Figure 7:**
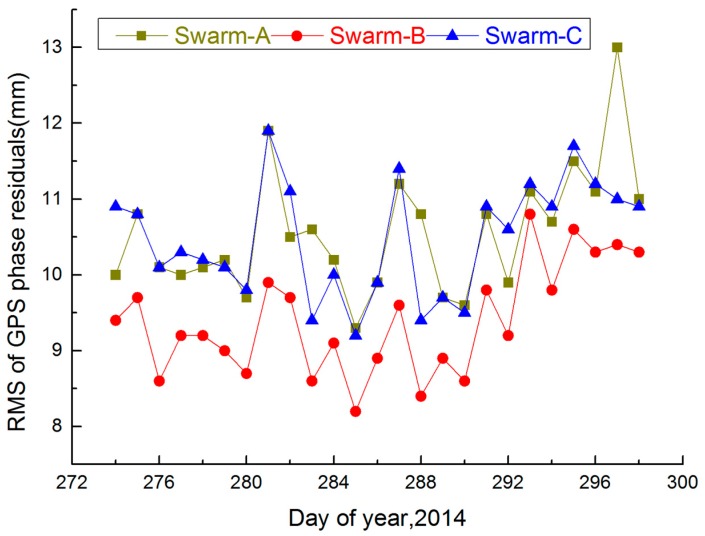
RMS values of GPS phase residuals for Swarm-A, Swarm-B and Swarm-C during day of year (DOY) 274–298, 2014.

Figure 7 summarizes the RMS of GPS phase residuals for Swarm satellites (Swarm-A, Swarm-B and Swarm-C), DOY 274–298, 2014. Table 2 shows the mean RMS of GPS phase residuals for Swarm-A, Swarm-B and Swarm-C, DOY 274–298, 2014. Figure 7 and Table 2 indicate that RMS of GPS phase residuals for Swarm-A is nearly equal to Swarm-C. The reasons may be that two Swarm satellites have the same orbit height and space environment. RMS of GPS phase residuals for Swarm-B is smaller than those of Swarm-A and Swarm-C. The reason may be that Swarm-B orbit height is higher than those of Swarm-A and Swarm-C, and force model errors (e.g., atmospheric drag and solar radiation pressure) are increased with the reduction of the orbit height. Moreover, the mean RMS of GPS phase residuals for Swarm-A, Swarm-B and Swarm-C range from 9–11 mm, which has a good consistency for each day during high ionospheric activity.

Therefore, the RMS of GPS phase residuals for Swarm-A, Swarm-B and Swarm-C are reasonable and stable by using the Swarm orbit strategy under high ionospheric activity.

### 4.2. Comparison with PSO Produced by ESA

Swarm PSOs are released by ESA, which are of a high accuracy, and are widely applied throughout the world [3]. Swarm ZD reduced-dynamic POD is compared with Swarm PSOs produced by ESA. The related results are as follows.

**Figure 8:**
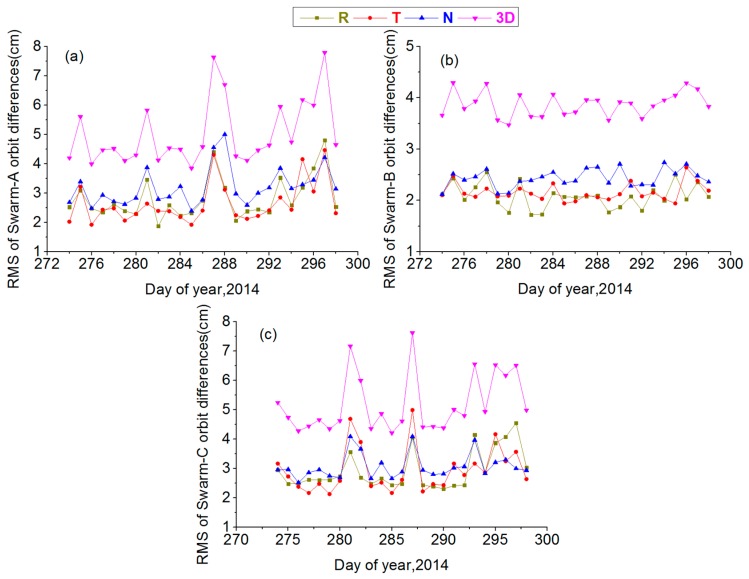
RMS values of orbit differences for Swarm-A, Swarm-B and Swarm-C in the radial (R), along-track (T) and cross-track (N) directions during DOY 274–298, 2014: (**a**) RMS values of Swarm-A orbit differences; (**b**) RMS values of Swarm-B orbit differences; (**c**) RMS values of Swarm-C orbit differences.

Figure 8a shows the RMS values of orbit differences for Swarm-A in R, T, N and 3D directions during DOY 274–298, 2014. The figure indicates that RMS values of orbit differences for Swarm-A in R, T and N directions are in the range of 2–5 cm, and 3D direction is in the range of 4–8 cm.

Figure 8b shows the RMS values of orbit differences for Swarm-B in R, T, N and 3D directions during DOY 274–298, 2014. The figure indicates that the RMS values of orbit differences for Swarm-B in R, T and N directions are within 1.5–3 cm, and 3D direction is within 3–4.5 cm.

Figure 8c shows the RMS values of orbit differences for Swarm-C in R, T, N and 3D directions during DOY 274–298, 2014. The figure indicates that RMS values of orbit differences for Swarm-C in R, T and N directions are in the range of 2–5cm, and 3D direction is within 4–8 cm.

Table 3 shows the RMS values of orbit differences for Swarm-A, Swarm-B and Swarm-C during DOY 274–298, 2014. The table indicates that the mean RMS values of orbit differences for all Swarm satellites are within 2–3 cm in R and T directions, within 2–4 cm in N directions, and in the range of 3–6 cm in the 3D direction.

Figure 8 and Table 3 indicate that orbit accuracy of Swarm-B is better than that of Swarm-A and Swarm-C in each direction, and orbit accuracy of Swarm-A is basically equal to that of Swarm-C. These results are consistent with the conclusion of Swarm GPS phase residuals. The precise orbits for all three Swarm satellites also have high and stable orbit quality on the basis of the Swarm orbit strategy in the article under high ionospheric activity.

**Figure 9:**
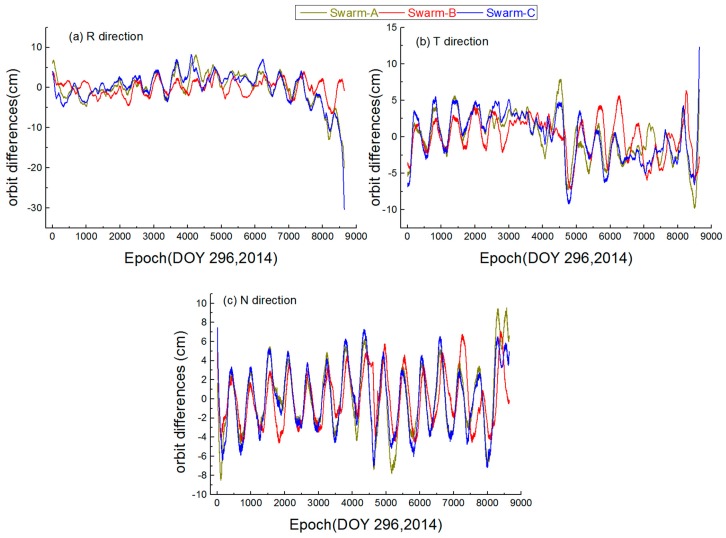
Orbit differences for Swarm-A, Swarm-B and Swarm-C in the R, T and N directions on the DOY 296, 2014.

For a more intuitive understanding of the single-arc orbit differences between the Swarm orbits with Swarm PSOs produced by ESA, we offer the orbit difference map of the day with the strongest ionospheric activity (DOY 296, 2014, F10.7 = 224.8 sfu), as shown in Figure 9. The figure shows the orbit differences for Swarm-A, Swarm-B and Swarm-C in the R, T and N directions on the DOY 296, 2014. Moreover, no significant systematic differences are found between the orbital results and PSOs produced by ESA. The orbit differences mainly come from the random error of Swarm orbital period, which agrees with the general law of reduced-dynamic OD. In addition, during the period of high ionospheric activity, space-borne GPS observation quality and force deficiencies are worse than normal. These factors lead to orbit differences a bit larger than usual. In conclusion, the results show that the Swarm orbit strategy is effective and offers good orbit quality under the highest ionospheric activity.

### 4.3. SLR Validation

Swarm-A/B/C satellites were not only equipped with a GPS receiver, but also with an array of SLR retro-reflectors. Thus, we can use high-precision SLR measurements to validate the Swarm reduced-dynamic orbits. Using the SLR measurements to validate the Swarm reduced-dynamic orbits, it is actually the difference between the SLR measurements and the distance between the SLR ground stations and the Swarm reduced-dynamic orbits. Moreover, the related corrections (e.g., tropospheric delay, tide correction, satellite center mass correction, and relativistic correction) to SLR measurements should be considered [35].

The Swarm ZD reduced-dynamic orbits were independently validated with SLR measurements over the same 25 days (days 274–298, 2014). SLR measurements which have been compressed in normal point are obtained from the EUROLAS Data Center (EDC). During the processing, related corrections are applied in SLR measurements. In addition, an elevation cut-off angle of 10° has been applied to all SLR ground stations and tropospheric delay has been modeled according to the International Earth Rotation Service (IERS) 2010 [29]. The coordinates of the SLR stations are based on the Station Location Reference Frame (SLRF) 2008 (https://ilrs.cddis.eosdis.nasa.gov/science/awg/SLRF2008.html). It is very difficult to track for the SLR ground station, because Swarm-A/B/C satellites fly fast at the altitude of 480–530 km. During this period, 1023 SLR residuals were computed using measurements from 10 SLR stations for Swarm-A, 3438 SLR residuals were computed using measurements from 15 SLR stations for Swarm-B, and 1045 SLR residuals were computed using measurements from 12 SLR stations for Swarm-C, respectively. See Table 4 for details.

**Figure 10:**
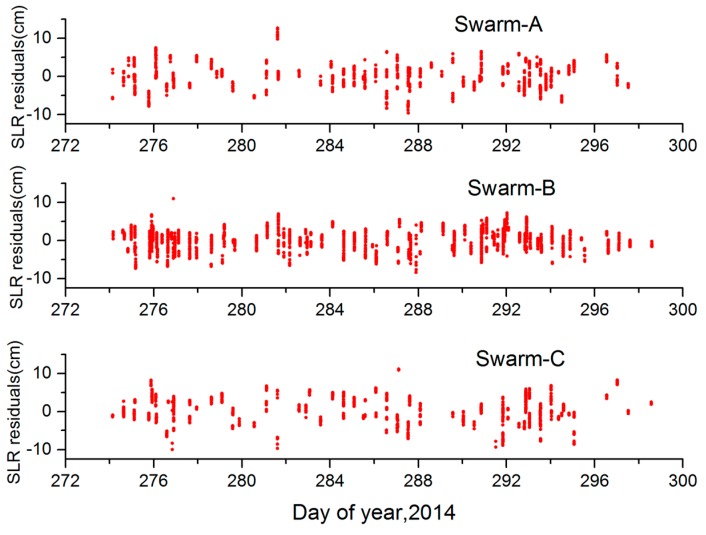
SLR residuals for the Swarm ZD reduced-dynamic POD during DOY 274–298, 2014.

SLR residuals of Swarm-A/B/C are summarized in Table 4 and Figure 10, respectively. From Table 4 and Figure 10, we can conclude that the RMS of SLR residuals for Swarm-A/B/C is 3.3 cm, 2.7 cm and 3.6 cm, respectively. In addition, the mean SLR residuals for Swarm-A/B/C is 0.05 cm, −0.01 cm and 0.14 cm, respectively. These results show that there is no significant bias in the Swarm reduced-dynamic POD, and the computed reduced-dynamic orbits for Swarm-A/B/C are of high quality under high ionospheric activity.

## 5. Conclusions

This study investigates how well Swarm orbits can be determined under high ionosphere activity using only space-borne GPS data and optimized pseudo-stochastic pulses based on the ZD reduced-dynamic method. The challenge is determining how to compute the Swarm orbits and access orbit quality. Therefore, the proper assessment of orbit quality, development of the proper strategies for Swarm POD, and optimization of pseudo-stochastic pulses are the main tasks of this study.

The precise orbits for all three Swarm satellites using real space-borne dual-frequency GPS data in the ZD reduced-dynamic method are computed under high ionospheric activity. The quality of the Swarm ZD POD is assessed by GPS phase residuals, comparison with Swarm PSOs released by ESA, and SLR validation. We draw the following conclusions based on the above assessment results.

(1) The chosen force models and observation models used in the reduced-dynamic determination have good fit under high ionospheric activity. The orbital fits to GPS tracking data for Swarm-B are better than those of Swarm-A and Swarm-C.
(2) Pseudo-stochastic pulses with a time interval of 6 min and a priori STD of 10^−2^ mm/s in the R, T and N directions are optimized in the Swarm ZD reduced-dynamic POD.
(3) The mean RMS values of orbit differences for all three Swarm satellites are within 2–4 cm in R, T and N directions and 3–6 cm in 3D direction. Independent SLR validation indicates that the accuracy of the Swarm reduced-dynamic orbits is in the range of 2–4 cm. Moreover, Swarm-B orbit accuracy is better than those of Swarm-A and Swarm-C. Therefore, no obvious systematic bias is found between PSOs produced by ESA and orbit solutions computed using the force models and orbit strategy.

## Figures and Tables

**Table 1:** The model standards adopted for Swarm zero-differenced (ZD) reduced-dynamic precise orbit determination (POD).

Model	Description
Force models	
Mean Earth gravity	EIGEN-6S4 (European Improved Gravity model of the Earth by New techniques) [16]
N-body	JPL DE405(Jet Propulsion Laboratory Solar System Ephemeris) [28]
Relativity	IERS 2010 (International Earth Rotation Service 2010) [29]
Solid-earth tides	IERS 2010 [29]
Ocean tides	FES2004(Modelling the global ocean tides: modern insights from FES2004) [30]
GPS observation models	
Space-borne GPS data	code and phase observation, 10 s sampling interval
GPS orbits	Center for Orbit Determination in Europe (CODE) final GPS precise orbit, 15 min sampling interval
GPS clock	CODE final precise clock, 5 s sampling interval
GPS phase model	igs08.atx
Elevation cut-off	3°
Estimated parameters	
Six initial conditions	$a,e,i,\Omega ,\omega ,T_{0}$
Swarm clock bias	Bias epoch-wise
Ambiguity parameter	ZD ambiguity estimation
Pseudo-stochastic pulses	time interval and a priori standard deviation (STD)

**Table 2:** Mean RMS values of GPS phase residuals for Swarm-A, Swarm-B and Swarm-C during DOY 274–298, 2014.

Satellite	Mean RMS Values of GPS Phase Residuals (mm)
Swarm-A	10.6
Swarm-B	9.4
Swarm-C	10.5

**Table 3:** Mean RMS values of orbit differences for Swarm-A, Swarm-B and Swarm-C during DOY 274–298, 2014.

Satellite	Mean RMS Values of Orbit Differences (cm)			
	R	T	N	3D
Swarm-A	2.81	2.64	3.20	5.03
Swarm-B	2.08	2.16	2.44	3.87
Swarm-C	2.94	2.95	3.08	5.20

**Table 4:** Satellite Laser Ranging (SLR) residuals for Swarm-A/B/C.

Satellite	Number of SLR Station	Number of Normal Point	Mean (cm)	RMS (cm)
Swarm-A	10	1023	0.05	3.3
Swarm-B	15	3438	−0.01	2.7
Swarm-C	12	1045	0.14	3.5
